# Refreshing Sleep and Sleep Continuity Determine Perceived Sleep Quality

**DOI:** 10.1155/2016/7170610

**Published:** 2016-06-16

**Authors:** Eva Libman, Catherine Fichten, Laura Creti, Kerry Conrod, Dieu-Ly Tran, Roland Grad, Mary Jorgensen, Rhonda Amsel, Dorrie Rizzo, Marc Baltzan, Alan Pavilanis, Sally Bailes

**Affiliations:** ^1^Jewish General Hospital, Montreal, QC, Canada H3T 1E2; ^2^McGill University, Montreal, QC, Canada H3A 0G4; ^3^Dawson College, Montreal, QC, Canada H3Z 1A4; ^4^University of Montreal, Montreal, QC, Canada H3T 1J4; ^5^Mount Sinai Hospital, Montreal, QC, Canada H4W 1S7; ^6^St. Mary's Hospital, Montreal, QC, Canada H3T 1M5

## Abstract

Sleep quality is a construct often measured, employed as an outcome criterion for therapeutic success, but never defined. In two studies we examined appraised good and poor sleep quality in three groups: a control group, individuals with obstructive sleep apnea, and those with insomnia disorder. In Study 1 we used qualitative methodology to examine good and poor sleep quality in 121 individuals. In Study 2 we examined sleep quality in 171 individuals who had not participated in Study 1 and evaluated correlates and predictors of sleep quality. Across all six samples and both qualitative and quantitative methodologies, the daytime experience of feeling refreshed (nonrefreshed) in the morning and the nighttime experience of good (impaired) sleep continuity characterized perceived good and poor sleep. Our results clarify sleep quality as a construct and identify refreshing sleep and sleep continuity as potential clinical and research outcome measures.

## 1. Introduction

Among the diagnostic criteria for insomnia disorder, the Diagnostic and Statistical Manual for Mental Disorders (DSM-5, [1]) invokes the term “sleep quality.” In clinical and research practice it is common to ask the patient or participant the following: “rate the quality of your sleep on a scale from 1 to 10” [2].

Sleep quality is a construct widely used, measured, and employed as an outcome criterion of therapeutic success [3–6], yet, it has never been actually defined [7, 8]. In practice, poor sleep quality is frequently presented as synonymous with sleep characteristics of insomnia: a collection of sleep measures such as difficulty getting to sleep and staying asleep and poor sleep efficiency [9–11]. It is also generally accepted that poor sleep quality is associated with impaired daytime functioning [12, 13]. Indeed, the Pittsburgh Sleep Quality Index (PSQI) [14], a widely used and accepted measure of global poor sleep quality, is based on an individual's appraisal of nocturnal and daytime* dysfunction*, effectively imposing the definition as well as the measurement.

What, then, is subjectively perceived* good* sleep quality? Commonly, it is believed to be the absence of those sleep-related difficulties seen in insomnia. Alternately, it is sometimes defined by greater well-being and better psychological functioning [15]. Although good sleep quality appears to be associated with a range of positive outcomes, how it is to be defined and measured is unclear. Notably, the commonly used sleep quality rating scale from 1 to 10 (e.g., [2, 16]) permits measurement in the positive range as well as the negative or intermediate one; however, we still have little idea of how the value assigned to sleep quality appraisal is derived. Is it based on nocturnal sleep-related experience? Is it interpreted on the basis of feeling rested in the morning or by more positive mood or more efficient functioning in the daytime? How might individual experience affect appraisal of sleep quality? It is not known whether the appraisal process among individuals with no particular sleep problem is similar to or different from that of individuals who perceive their sleep as problematic (e.g., those with insomnia or where there is a physical disorder present that regularly arouses the individual from sleep, such as obstructive sleep apnea (OSA)).

We argue that “sleep quality” is a term loosely constructed and vaguely defined by clinicians and researchers while having little idea of what it means to the average person. Harvey and colleagues [12] took the unusual step of asking individuals who were not researchers or clinicians, in a “speak freely” format, to describe what sleep quality meant to them. These participants were relatively young individuals and the study provides some insight into what defines sleep quality for this population. The present investigation was designed to add another piece to the puzzle by investigating the meaning of sleep quality in a wider range of samples and ages, including some more clinically representative (i.e., older individuals with chronic insomnia and patients with sleep disorder, typically seen in primary care and sleep clinics).

Specifically, in Study 1 we used open-ended methodology to examine how both good and poor sleep quality are appraised in three groups of individuals: those with insomnia disorder, those with OSA, and those with no specific sleep-related complaint. Next, in Study 2, we amplify these findings using closed-ended measures. We again administered measures to individuals with insomnia disorder, with OSA, and with no specific sleep-related complaints who were recruited from different populations and who did not participate in Study 1. The purpose was to examine daytime and nighttime correlates and predictors of sleep quality in these varied clinical and community groups. Results from Studies 1 and 2 permit a more representative picture than that which has so far been presented in the literature.

The importance of the question as to the meaning of sleep quality is that, without a commonly agreed upon language, researchers may base conclusions on incongruent measures; clinicians may unwittingly set therapy outcome goals discrepant from those of their patients.

## 2. Method

### 2.1. Measures

#### 2.1.1. Questionnaires

*Open-Ended Sleep Quality Measure*. Two questions were presented to participants on a single page: “How do you tell whether you've had a good night's sleep?” and “How do you tell whether you've had a poor night's sleep?” The questions were presented in counterbalanced order and preceded by the statement, “Everyone has experienced good and poor sleep.” Responses were coded by two trained coders into 21 good sleep quality and 21 corresponding poor sleep quality categories in accordance with a coding manual [17]; category descriptions and examples are available in Table 1. Scores are evaluated in two ways: to evaluate the broad categories we summed the total number of* responses* participants provided in the good and poor sleep daytime and nighttime categories. To examine the popularity of each category in Table 2 we evaluated the number of* participants* who had at least one response in the category. The coding manual consists of inductively derived categories based on participants' responses. Coders were trained to a minimum of 70% interrater agreement. Spot checks of interrater agreement all exceeded 70%.

*Sleep Questionnaire [16, 18]*. This brief retrospective measure inquires about typical nocturnal and daytime (e.g., fatigue, sleepiness, and difficulty concentrating) experiences during the past month and the frequency (0–7 days/week) of nonrefreshing sleep and difficulty falling asleep and getting back to sleep after nocturnal awakenings, as well as the typical duration of total nocturnal sleep time (TST), sleep onset latency (SOL), and wake after sleep onset (WASO). The measure also asks about typical sleep quality (10-point scale), the presence of insomnia (yes/no), and the duration of the sleep problem as well as distress related to it. Validation for the Sleep Questionnaire shows good test-retest reliability (*r* values range from .58 to .92) [16] and high correlations between equivalent scores on this measure and a daily sleep diary (e.g., *r* = .83, .64, and .69 for TST, SOL, and WASO, resp. [19]). We used the sleep quality item (1 = very poor, 10 = very good) from this measure as the predicted variable when correlates and predictors were examined.

In accordance with established practice, self-report rather than polysomnography was used to diagnose insomnia [20]. Information provided by the Sleep Questionnaire allowed us to diagnose the presence of difficulty initiating or maintaining sleep (DIMS) and of insomnia disorder in accordance with DSM-5 criteria [1].

*Sleep Symptom Checklist (SSC) [21].* The SSC is a 21-item survey of a broad range of symptoms that are both directly and indirectly related to sleep disorders. Respondents rate each symptom, including poor sleep quality, for its severity from 0 (not at all) to 3 (very severe) based on the previous month. In this measure, sleep quality is evaluated by severity of poor sleep quality (0 = not at all severe, 3 = very severe).

#### 2.1.2. Diagnosing Presence and Absence of OSA

*Polysomnography (PSG)*. Nocturnal PSG was used to obtain sleep parameter scores (i.e., frequency of nocturnal arousals, total sleep time, sleep onset latency, wake after sleep onset, and sleep efficiency) as well as OSA related factors.

*Home Sleep Oximetry Assessment*. A SnoreSat Recorder (SegaTech Electronics Inc., Calgary, Canada) was used to screen healthy control group participants for the presence of OSA [22, 23].

### 2.2. Study 1

#### 2.2.1. Method

This study used an open-ended qualitative approach to gather written descriptions of what constitutes both poor and good sleep quality by individuals with OSA and individuals with and without insomnia.

Three groups included 49 individuals seeking or already enrolled in cognitive-behavior insomnia therapy (CBT-I) (16 male, 33 female, mean age = 46, range = 18–83, median = 48), 27 individuals with OSA (12 male, 15 female, mean age = 58, range = 40–83, median = 55), and a convenience sample of 45 non-treatment-seeking individuals (Normal Sleeper Control Group: 15 male, 29 female (1 participant did not indicate gender), mean age = 47, range = 19–79, median = 45). None of the participants in the control group met the diagnostic criteria for insomnia disorder. There was no significant difference in gender composition among the three samples; participants in the OSA sample were significantly older (mean = 58.64) than those in the other two groups (Control, *M* = 47.36, CBT-I *M* = 47.48), *F*(2,118) = 5.88, *p* = 0.004.

Participants in the OSA group had undergone nocturnal polysomnography. The minimum apnea/hypopnea index (AHI) was 10 and they had been diagnosed with OSA by a sleep specialist. Participants in the other two groups had not been evaluated for OSA.

Those in the CBT-I group were mainly self-referred to a sleep clinic for a complaint of insomnia. Participants in the CBT-I group had experienced insomnia for a mean of 10 years (SD = 13, range = 1–50 years, median = 4 years). They experienced insomnia in an average of 5 nights per week and took sleep medication in a mean of 4 nights per week. On a 10-point insomnia distress scale the CBT-I group's mean score was 8.14 (SD = 1.68, range = 4–10).

All participants completed the paper-and-pencil Sleep Questionnaire and the Open-Ended Sleep Quality Measure (counterbalanced order of good and poor sleep quality). Participants could take as much time as they wished.

#### 2.2.2. Results

*Differences among the Samples*. A multivariate analysis of covariance comparison on selected items on the Sleep Questionnaire showed a significant difference among the three groups, *F*(22,176) = 3.15, *p* < 0.001. Age was the covariate. Univariate analysis of covariance tests revealed significant differences for all of the variables (i.e., SOL, waking after sleep onset, waking too early, sleep quality, sleep satisfaction, feeling refreshed in the morning, nocturnal tension, daytime fatigue, sleepiness, and difficulty concentrating). The Tukey HSD test showed that the control and CBT-I groups were significantly different from each other in all cases. The mean for the OSA group fell between these in all comparisons, sometimes differing significantly from both groups, sometimes from one or the other.

**Figure 1:**
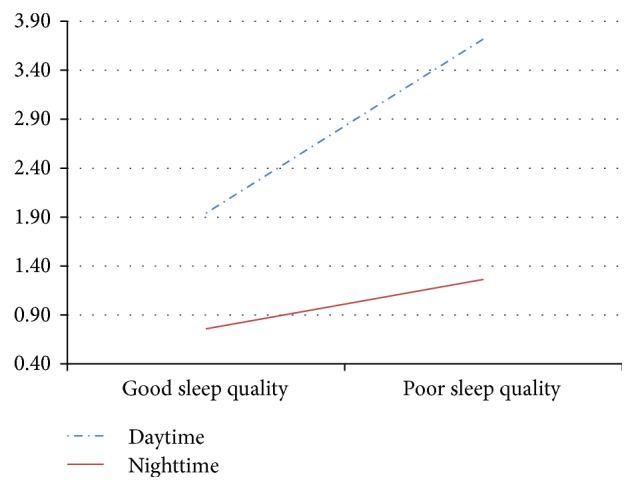
Interaction of time of day by valence for number of different response categories. Scores are based on the total number of responses participants provided.

*Sleep Quality*. A series of between groups ANOVAs comparing the 3 groups on the summed total number of responses participants provided in the good and poor sleep daytime and nighttime scores showed a significant finding only on daytime poor sleep quality, *F*(1,121) = 36.33, *p* < 0.001. The Tukey HSD test showed that the OSA group had significantly more responses in this category than those with insomnia, who, in turn, had significantly more responses than the control group. A repeated measures ANOVA showed a significant main effect for time of day, *F*(1,120) = 115.94, *p* < 0.001; as might be expected, more daytime than nocturnal responses were reported. There was also a significant main effect for valence, *F*(1,120) = 159.23, *p* < 0.001, with more poor than good sleep quality related experiences reported. What is of special interest is the significant interaction of valence × time of day, *F*(1,120) = 35.85, *p* < 0.001. There were a disproportionately large number of responses in the daytime poor sleep quality category, best seen in Figure 1.

Table 2, where we provide information of the percentage of participants who had at least one response in each category, further illustrates these results showing the larger variety of poor sleep quality than good sleep items. Although there were subtle differences among the three groups, Table 2 also demonstrates that the top four categories are common to both good and poor sleep quality for all groups. These are feeling refreshed/unrefreshed in the morning, good/poor sleep continuity, fatigue/energy, and good/bad mood.

#### 2.2.3. Discussion: Study 1

We expanded on Harvey et al.'s [12] procedure comparing people with insomnia disorder, those with OSA, and normal controls on the meaning of sleep quality and determining aspects of perceived sleep quality. As Harvey et al. did, we required participants to generate responses. However, it is notable that, unlike Harvey et al.'s samples, our participants with insomnia met diagnostic criteria for the disorder and were treatment-seeking; our control group represented a wide age range, and we added a comparative sample of individuals with obstructive sleep apnea.

In view of these methodological differences, it is noteworthy that our findings are similar to Harvey et al.'s [12]: sleep quality is similarly defined by those with and without sleep problems. Specifically, we found (a) the meaning of sleep quality, both good and poor, is generally similar among individuals with OSA as well as with and without insomnia, and (b) daytime and negative experiences appear to be the most important bases for judging sleep quality. However, it may be that people have a much richer vocabulary for describing daytime consequences than for describing nighttime problems, possibly because nighttime experience is less accessible to consciousness. Our findings add to the understanding of sleep quality as a construct by showing the following: (1) More people reported aspects of poor than of good sleep quality. (2) It is poor sleep quality that was more likely to be reflected in how people feel during the day compared to the night. (3) Individuals used more descriptive aspects when it came to poor sleep, as compared to good sleep. (4) The most important nighttime experience characterizing good sleep quality was infrequent awakenings (i.e., good sleep continuity). (5) The most frequent concepts used to characterize good sleep quality were feeling refreshed in the morning, infrequent nocturnal awakenings, feeling energetic and in a good mood, and good daytime functioning. (6) Poor sleep quality was characterized by the absence of positive daytime aspects (e.g., not feeling refreshed in the morning) as well as by fragmented sleep during the night (i.e., poor sleep continuity). In addition, poor sleep quality was characterized by inadequate amount of sleep, light sleep, feeling tired, being in a bad mood, experiencing morning headache, poor daytime functioning, feeling sleepy, and poor concentration, along with aches and pains during the day.

### 2.3. Study 2

#### 2.3.1. Overview

The purpose of this study was to validate the findings from Study 1 using a large existing database that included self-report sleep measures, polysomnography and home sleep evaluations, and measures of daytime and psychological functioning. Among the questionnaire items were two that examine good and poor sleep quality (paralleling, in closed-ended fashion, these aspects explored by open-ended format in Study 1): from the Sleep Questionnaire [16], the item “Generally, what is the quality of your sleep?” rated from 1 (very poor) to 10 (very good); from the Sleep Symptom Checklist [21] the symptom severity rating of poor sleep quality is from 0 (not at all severe) to 3 (very severe). Other items in these two questionnaires were analyzed to determine which single item or combination of items best predict ratings of sleep quality. Consistent with Study 1, we selected two clinical groups (OSA and CBT-I) as well as individuals with no sleep-related complaints (control group) for analyses.

#### 2.3.2. Method

The total number of participants was 171; none participated in Study 1. This included 88 primary care patients recently diagnosed with OSA (47 male, 41 female; mean age = 57, range = 40–89, median = 55), 57 patients seeking CBT-I (17 male, 40 female; mean age = 46, range = 20–80, median = 44), and 26 community participants (control group) (10 male, 16 female; mean age = 48, range = 22–71, median = 49) with no OSA or insomnia. A univariate analysis of variance comparing age showed that the OSA group was significantly older than the other two groups who did not differ (*F*(2,169) = 10.9, *p* < 0.001).

OSA participants were primary care patients aged 40 and older who were willing to undergo one night of polysomnography in a sleep lab and who had an apnea/hypopnea index or respiratory disturbance index (AHI/RDI) ≥ 10, mean severity = 32.9, SD = 22.9. Patients with current severe medical or psychiatric disorder were excluded as well as those who had restless legs syndrome (RLS) or periodic limb movement disorder (PLMD).

The CBT-I group consisted of consecutive patients seeking cognitive-behavioral treatment for insomnia at a hospital based CBT clinic. The majority (80%) were referred by sleep clinics, having tested negative for other sleep disorders. The rest were self-referred; those with known OSA, RLS, or PLMD were excluded. They experienced insomnia, an average of 5.6 (SD = 1.6) nights per week, and took sleep medication, a mean of 3.4 (SD = 3.1) nights per week. The mean duration of insomnia was 9.8 (SD = 11.8) years and the mean reported distress (1–10) was 8.3 (SD = 1.8).

The control group participants were community volunteers screened for OSA using the home monitoring device (AHI ≥ 10). None met criteria for insomnia.

#### 2.3.3. Results

To reduce the number of variables into more coherent dimensions, a principal components factor analysis with varimax rotation was performed on the entire Study 2 sample for 34 individual items on the Sleep Questionnaire and the SSC, excluding the two sleep quality items of interest. Ten items were eliminated since they failed to load consistently within a single factor or were conceptually at odds with other items within a factor. The optimal rotated solution yielded 5 factors, based on 24 items: 11 from the Sleep Questionnaire and 13 from the Sleep Symptom Checklist. Two items, “refreshed in the morning” and “sleep is non-refreshing,” were found to have weak loadings on all factors. In addition, their presence appeared to weaken the factor loadings of two other variables. Therefore, they were removed from the factor analysis and treated as a separate subscale. The final factor-based subscales, presented in Table 3, were assigned descriptive labels as follows: daytime difficulty, sleep initiation problem, sleep maintenance problem, psychological distress, OSA symptoms, and non-refreshed in the morning.

Subscale scores, based on the 6 factors, were calculated by converting raw item scores to standardized *z*-scores and summing the *z*-scores within each factor, taking into account the factor loading valence. Univariate ANOVAs and Bonferroni post hoc tests were conducted comparing the 3 groups on each of the 6 subscale scores. The *F* tests were significant for all subscales (*p* < 0.0001). The pattern of differences among groups was consistent with their diagnostic characteristics. For the subscale sleep initiation problem, *F*(2,149) = 90.70, *p* < 0.001, sleep maintenance problem, *F*(2,145) = 33.69, *p* < 0.001, and psychological distress, *F*(2,158) = 21.00, *p* < 0.001, the pattern of group differences was identical: the CBT-I group was significantly worse than the others, followed by the OSA group, which was significantly worse than the control group. On the daytime difficulty subscale, the CBT-I group had significantly worse scores than the OSA and control groups, which did not differ from each other, *F*(2,158) = 22.27, *p* < 0.001. On the sleep disorder subscale, the OSA group was significantly worse than either the CBT-I or control group, who did not differ significantly from each other, *F*(2,143) = 28.93, *p* < 0.001. Finally, on the non-refreshed in the morning subscale, the CBT-I group had the worst scores, followed by the OSA group, which was, in turn, worse than the control group, *F*(2,159) = 44.45, *p* < 0.001. All post hoc differences were significant at the .05 level after adjustment for multiple comparisons.

Next, regression analyses were carried out separately for the three groups to determine which subscales best predicted sleep quality. Predicted variables were sleep quality on the Sleep Questionnaire (i.e., “Generally, what is the quality of your sleep?” (1 = very poor, 10 = very good)) and severity of* poor* sleep quality on the Sleep Symptoms Checklist (i.e., “Rate how severe this was during the past month”:* poor* sleep quality (0 = not at all severe, 3 = very severe)). Results in Table 4 show that when predicting sleep quality on both measures, the sleep maintenance and non-refreshed in the morning subscales were important predictors for all three groups. For the control group only, the sleep quality measure (1–10) did not have significant predictors among the subscales.

#### 2.3.4. Discussion: Study 2

Results from Study 2 show that closed-ended questionnaire responses from clinical and community samples were consistent with the open-ended responses of Study 1 participants. What was immediately evident from the results is that daytime and nighttime sleep-related experiences loaded on separate factors. Interestingly, sleep initiation and sleep continuity, variables often associated with insomnia, appeared as separate constructs. Of these two constructs, only the experience of sleep continuity was found to be linked to the perception of sleep quality. In fact, neither sleep initiation nor psychological distress, two hallmarks of insomnia disorder, was predictive of sleep quality.

Using a very different methodology from Study 1, we found that feeling refreshed in the morning and sleep continuity at night were the key predictors of the perception of both good and poor sleep quality. By and large, this relationship was found to be consistent among very different participant groups: those with insomnia, those with OSA, and those with no sleep problems at all. The lesser importance of daytime experience found here compared to Study 1 could be due to the limitation of the close-ended questionnaire and the bias toward symptom-based questions rather than positive experience.

Secondarily, the development of subscales allowed us to examine similarities and differences among the various clinical and community groups and may provide a “backdrop” from which to interpret the predictors. For example, although insomnia-related subscales showed diagnostically appropriate differences among the three groups, only sleep maintenance (i.e., sleep continuity) was found to be predictive of sleep quality ratings.

## 3. General Discussion

The findings of the two studies show remarkable consistency in appraisals of sleep quality across the diverse samples. Specifically, the most important components in all cases were feeling refreshed in the morning and good sleep continuity, which characterized perceived good sleep quality, and feeling nonrefreshed in the morning and impaired nocturnal sleep continuity, which characterized perceived poor sleep quality. This was true regardless of whether we used qualitative or quantitative measures and regardless of characteristics of the samples: “normal” sleepers, those with insomnia disorder, and those with OSA.

In addition, in Study 1, where a sample of mostly middle-aged participants including “normal” sleepers, those with chronic insomnia, and those with OSA, were asked to define sleep quality in their own words, we demonstrated that (a) people were more inclined to describe aspects of poor than of good sleep quality, and (b) descriptors were more extensive for daytime as compared to nighttime aspects of sleep quality; this was especially true for poor sleep quality. The qualitative data also yielded descriptors not reflected in existing sleep quality measures (e.g., “clear headed,” “morning headache,” “good/poor mood,” and “no bags under the eyes”). These findings remind us that individual differences in the perception and interpretation of good and poor sleep quality might be usefully incorporated in therapeutic evaluation. The present results confirm and elaborate on Harvey et al.'s [12] results, where the samples were much younger and where individuals with OSA were not included.

In Study 2, where quantitative data were examined by factor analysis and regression equations, the results also show two distinct clusters related to insomnia: a factor related to sleep continuity and one related to sleep initiation. This suggests that these two hallmarks of the insomnia complaint may have different correlates and, perhaps, etiologies.

### 3.1. Limitations

A limitation of this research may be that for Study 2 we used a data set not specifically designed to evaluate the question of how sleep quality is determined. Therefore, there is some inconsistency among the measures used. We believe, however, that this compromised methodological rigor is outweighed by the wealth of information afforded by the novel comparisons we were able to provide, that is, “ecologically valid samples,” with and without various kinds of sleep disorders over a wide age range.

### 3.2. Conclusions

The findings suggest that the construct of sleep quality has a common core; perceived good and poor sleep quality have distinct characteristics with definable components that are common to diverse groups. In addition, there is a defined “universe” of components that comprise the individual differences among different groups. An important contribution of the present exercise is that the findings confirm (a) that the concepts of “good sleep quality” and “poor sleep quality” are not simply polar opposites, (b) that measures such as the Pittsburgh Sleep Quality Index [14] measure not sleep quality but sleep* difficulty*, (c) that sleep quality has a consistent meaning in individuals' actual experience, and (d) that the data provide a rationale for the two most important sleep quality components, feeling (non)refreshed in the morning and nocturnal sleep continuity, to be used as outcome criteria in research and clinical practice.

## Figures and Tables

**Table 1:** Open-ended codes in alphabetical order with examples.

Good sleep examples	Category name	Poor sleep examples
When I wake up and am not groggy	Body warmth	I feel my body is too warm
	Clear headed/groggy	Not thinking clearly, feel fuzzy-headed and foggy when I wake up
	Clock watching	I looked at the clock a lot during the night
A good level of concentration	Concentration/attention	I have trouble concentrating and make mistakes
No dark circles under my eyes	Dark circles under eyes	Dark circles under my eyes
I function well throughout the day	Daytime functioning	When I can't do what I do on a daily basis
I feel I was in a deep sleep, no drifting in and out of sleep	Depth of sleep	My sleep was light, I feel I spent most of the night awake
My mind is caught up in the remainders of a super dream	Dreams	My mind is confused by bad dreams
If I am awake no earlier than at 6:30 it is great	Early wakening	I am awake several hours before sunrise
I feel energetic during the day	Fatigue/energy	I feel tired and lethargic during the day
No headache	Headache	I wake up with a headache
My memory is good	Memory	I'm forgetful
I'm in a good mood	Mood	I'm cranky and moody
My body is without pain	Pain/ache	I wake up and my body aches
I slept throughout the night, no night-time awakenings	Sleep continuity	I recall waking several times during the night
I don't feel sleepy at all	Sleepiness	Sometimes I nod off at my desk
I fall asleep when my head hits the pillow	Sleepy onset latency (SOL)	If it has taken me a long time to fall asleep
My mind is relaxed	Thoughts	I keep worry about how I am going to feel tomorrow
The less tossing and turning I've done the better	Tossing and turning	I noticed I was tossing and turning, couldn't find a place for myself
If I slept for 6+ hours	Total sleep time (TST)	If I don't get my minimum number of hours
I wake up easily and feel ready to get up	Waking feeling refreshed/non-refreshed	I feel exhausted when I wake up and feel like I still need rest

**Table 2:** Rank order of good and poor sleep quality categories in Study 1: percentages based on the number of participants who had at least one response in the category.

Rank		Good sleep quality						Poor sleep quality				
		Insomnia	OSA	Control	Total	Night versus day		Insomnia	OSA	Control	Total	Night versus day
1	**Refreshed in morning**	55%	41%	51%	**50%**	**Day**	**Frequent awakenings**	45%	22%	44%	**40%**	**Night**
2	**Infrequent awakenings**	41%	26%	31%	**35%**	**Night**	**Not refreshed in morning**	35%	37%	42%	**39%**	**Day**
3	**Lots of energy**	33%	44%	31%	**35%**	**Day**	**Tired**	43%	37%	36%	**39%**	**Day**
4	**Good mood**	27%	15%	22%	**22%**	**Day**	**Bad mood**	29%	26%	16%	**23%**	**Day**
5	Good daytime functioning	20%	11%	16%	17%	Day	Headache in morning	25%	15%	13%	18%	Day
6	Clear headed	10%	0%	11%	8%	Day	Inadequate TST	16%	7%	16%	14%	Night
7	Enough TST	4%	7%	11%	7%	Night	Poor daytime functioning	16%	11%	11%	13%	Day
8	Deep sleep	8%	4%	7%	7%	Night	Sleepy	16%	11%	9%	12%	Day
9	Good dreams	4%	7%	7%	6%	Night	Light sleep	16%	7%	9%	12%	Night
12	Good concentration	6%	4%	4%	5%	Day	Poor concentration	14%	4%	11%	11%	Day
21	Short SOL	6%	7%	0%	4%	Night	Aches and pains	10%	11%	9%	10%	Day
10	No aches and pains	4%	0%	2%	3%	Day	Waking too early	12%	0%	11%	9%	Night
11	Waking early	2%	0%	4%	3%	Night	Groggy	10%	0%	11%	8%	Day
13	Not sleepy	4%	7%	0%	3%	Day	Long SOL	10%	4%	9%	8%	Night
18	No headache in morning	4%	4%	0%	3%	Day	Disturbing thoughts	12%	7%	4%	8%	Day
15	Good memory	2%	0%	2%	2%	Day	Lots of tossing and turning	4%	0%	9%	5%	Night
17	Pleasant thoughts	2%	0%	2%	2%	Day	Bad dreams	2%	4%	9%	5%	Night
14	No dark circles under eyes	0%	0%	2%	1%	Day	Poor memory	2%	11%	2%	4%	Day
16	No tossing and turning	0%	0%	2%	1%	Night	Body too warm	6%	0%	0%	3%	Night
19	Body warm	0%	0%	0%	0%	Night	Dark circles under eyes	2%	0%	2%	2%	Day
20	No clock watching	0%	0%	0%	0%	Night	Clock watching	0%	0%	4%	2%	Night

*Note*. Bold font items were endorsed by over 20% of participants and are the top 4 categories for all three groups.

**Table 3:** Factor loadings of 34 Sleep Questionnaire and Sleep Symptom Checklist items.

	Daytime difficulty	Sleep initiation problem	Sleep maintenance problem	Psychological distress	Sleep apnea symptoms	Non-refreshed in morning
Sleepy during the day? (10 = very)	.877					
Tired during the day? (10 = very)	.821					
Daytime sleepiness (3 = very severe)	.807					
Daytime fatigue (3 = very severe)	.739					
Falling asleep during the day when not wanted (3 = very severe)	.652					
Naps, days/wk.	.576					
Difficult to concentrate? (10 = very)	.557					
Trouble falling asleep (3 = very severe)		.774				
Insomnia distress (10 = very)		.729				
Insomnia (3 = very severe)		.726				
Do you have insomnia? (yes/no)		−.708				
Tension when falling asleep (100 = very tense)		.696				
SOL (hrs.)		.618				
WASO (hrs.)			.790			
TST (hrs.)			−.723			
Waking up and trouble getting back to sleep (3 = very severe)			.689			
Waking up too early in the morning (3 = very severe)			.576			
Depression (3 = very severe)				.781		
Poor emotional well-being (3 = very severe)				.781		
Anxiety (3 = very severe)				.710		
Interruption of breathing during sleep (3 = very severe)					.853	
Snoring (3 = very severe)					.767	
Waking with a dry mouth (3 = very severe)					.587	
Refreshed in the morning (10 = very refreshed)						n/a
Sleep is non-refreshing (3 = very severe)						n/a

**Table 4:** Predicting sleep quality: study-wise linear regression.

	*β*	*p*	*R* ^2^
Sleep quality (1 = very low, 10 = very high)^1^			
OSA (*n* = 88)			
Predictors			
Constant	5.12	0.000	.47
Sleep maintenance	−.31	0.000	
Non-refreshed in the morning	−.59	0.000	
CBT-I (*n* = 57)			
Predictors			
Constant	4.53	0.000	.23
Non-refreshed in the morning	−.56	0.003	
Sleep maintenance	−.23	0.009	
Controls (*n* = 26)			
Predictors			
ns		ns	

Severity of *poor* sleep quality (0 = not at all severe, 3 = very severe)^2^			
OSA (*n* = 88)			
Predictors			
Constant	1.87	0.000	.36
Sleep maintenance	.16	0.001	
Non-refreshed in morning	.20	0.031	
CBT-I (*n* = 57)			
Predictors			
Constant	1.94	0.000	.41
Non-refreshed in morning	.48	0.000	
Controls (*n* = 26)			
Predictors			
Constant	2.23	0.000	.52
Sleep maintenance	.32	0.003	
Non-refreshed in morning	.28	0.026	

^1^Sleep Questionnaire.

^2^Sleep Symptoms Checklist.
